# Towards Optimization of Surface Roughness and Productivity Aspects during High-Speed Machining of Ti–6Al–4V

**DOI:** 10.3390/ma12223749

**Published:** 2019-11-14

**Authors:** Adel T. Abbas, Neeraj Sharma, Saqib Anwar, Faraz H. Hashmi, Muhammad Jamil, Hussien Hegab

**Affiliations:** 1 Department of Mechanical Engineering, College of Engineering, King Saud University, P.O. Box 800, Riyadh 11421, Saudi Arabia; faraz.mjct@gmail.com; 2 Department of Mechanical Engineering, Maharishi Markandeshwar (Deemed to be University), Mullana 133207, India; neeraj.sharma@live.com; 3 Industrial Engineering Department, College of Engineering, King Saud University, P.O. Box 800, Riyadh 11421, Saudi Arabia; sanwar@ksu.edu.sa; 4 College of Mechanical and Electrical Engineering, Nanjing University of Aeronautics and Astronautics, Nanjing 210016, China; engr.jamil@nuaa.edu.cn; 5 Mechanical Design and Production Engineering Department, Cairo University, Giza 12613, Egypt; hussien.hegab@uoit.ca

**Keywords:** high speed machining, titanium alloys, surface roughness, productivity, optimization

## Abstract

Nowadays, titanium alloys are achieving a significant interest in the field of aerospace, biomedical, automobile industries especially due to their extremely high strength to weight ratio, corrosive resistance, and ability to withstand higher temperatures. However, titanium alloys are well known for their higher chemical reactive and low thermal conductive nature which, in turn, makes it more difficult to machine especially at high cutting speeds. Hence, optimization of high-speed machining responses of Ti–6Al–4V has been investigated in the present study using a hybrid approach of multi-objective optimization based on ratio analysis (MOORA) integrated with regression and particle swarm approach (PSO). This optimization approach is employed to offer a balance between achieving better surface quality with maintaining an acceptable material removal rate level. The position of global best suggested by the hybrid optimization approach was: Cutting speed 194 m/min, depth of cut of 0.1 mm, feed rate of 0.15 mm/rev, and cutting length of 120 mm. It should be stated that this solution strikes a balance between achieving lower surface roughness in terms of R_a_ and R_q_, with reaching the highest possible material removal rate. Finally, an investigation of the tool wear mechanisms for three studied cases (i.e., surface roughness based, productivity-based, optimized case) is presented to discuss the effectiveness of each scenario from the tool wear perspective.

## 1. Introduction

Nowadays, titanium alloys are achieving a significant interest in the field of aerospace, biomedical, automobile industries especially due to their extremely high strength to weight ratio, corrosive resistance and ability to withstand higher temperatures [1]. However, titanium alloys are well known for their higher chemical reactive and low thermal conductive nature which, in turn, makes it more difficult to machine compared to steel [2]. The open literature showed that numerous researchers have investigated the machinability behavior of titanium alloys, mostly under the cutting speed under 100 m/min [1,2,3,4]. They found that high cutting temperature, major tool degradation, poor surface quality, and high cutting forces were the major obstacles during machining of titanium-based alloys [3]. Decades ago, Komanduri et al. [4] conducted experiments at lower cutting speed as well as higher cutting speed, and stated that during the titanium alloy machining, an increase in temperature results in the declining of tool life, as the temperature allows thermal expansion of tool and material of the workpiece as a result, a poor surface finish is obtained. Extensive research has been undertaken to study problems, such as tool wear, poor surface finish, and waviness incurred during the machining of these alloys. Sun et al. [5], for the end milling process, indicated that the surface roughness of Ti–6Al–4V alloy increased with the increase in depth of cut and feed. Ginting and Nouari [6] in their research work, employing cutting speed with the upper limit at 125 m/min, performed machining on titanium alloys using coated and uncoated carbide tools. It was observed that the surface quality of the workpiece material was better when an uncoated carbide tool was used. They also indicated that the surface roughness is dependent on the feed rate and cutting speed. Moreover, a research work performed on finish turning operation on titanium alloys proclaimed that the type of tool used and cutting feed rate have significant consequences on surface roughness [7]. Pervaiz et al. [8] commented that machining of Ti alloy generates a lower cutting force, which in turn, reduces power consumption, but increases the rate tool wear. Though the machining at lower to medium cutting speed showed good performance, in terms of productivity, it is not economically beneficial. As such, an attempt was taken by researchers to operate machines at higher cutting speeds, giving rise to a special technique called high-speed machining (HSM).

In recent years, HSM has played a prominent role in machining processes due to its low machining cost, good surface finish, and high material-removing capacity [9]. D’Mello et al. [10] performed high-speed turning on Ti–6Al–4V. The results indicated that the surface roughness of the workpiece is directly related to the cutting tool vibrations. Moreover, Ezugwu et al. [11] utilized pressurized coolant throughout the high-speed machining process of titanium alloys such as Ti–6Al–4V. The outcomes of these were compared with the conventional coolant supply and determined that with the increase in the coolant pressure, hardening effect on the machined surface was reduced. Studies of [12,13] compared and quantified the performance of coated carbide tool and uncoated carbide tool during turning operations. In both the research cases, the wear resistance was higher for coated carbide tool when collated with an uncoated carbide tool. Sun et al. [14] performed turning operation on Ti–6Al–4V alloy, at cutting speeds of 150 and 220 m/min, via a coated carbide insert, and examined the tool wear effect on cutting forces at high speeds. The results indicate that the tool wears dramatically exceeded and increased the forces. Another research study depicted that machining parameters such as tool vibrations, cutting speed, coolant usage, feed rate depth of cut, etc., have a major effect on the surface quality of titanium alloys [15]. Mello and Pai [16] investigated that, the surface roughness parameters R_a_ and R_t_ increase within tool vibrations and feed rate while the upsurge in cutting speed and tool wear decrease the R_a_ and R_t_. Another researcher Ramesh [17] indicated that in the case of titanium alloys such as Grade-5 are used, the feed rate plays a prominent role in surface roughness of the workpiece material. With different layouts of operations and various types of assistance to experiments, scholars reported benefits in machining overcoming challenges in HSM. However, one issue still remains to be studied: The tool wear during HSM.

Moreover, the employment of advanced optimization in key manufacturing engineering problems [18,19,20], specifically involving multiple inputs and outputs with non-linear relations amongst themselves, are found highly effective thanks to the advanced algorithms and computation science. Hence, optimization of high-speed machining responses of Ti–6Al–4V has been investigated in the present study using a hybrid approach of multi-objective optimization based on ratio analysis (MOORA) integrated with regression and particle swarm approach (PSO). This optimization approach is employed to offer a balance between achieving better surface quality with maintaining an acceptable material removal rate level. High-speed machining of titanium alloys upsurges cutting temperature that ruins the tool life and accelerates tool wear. Hence, flood cooling medium is utilized in the present study to tackle high cutting temperatures. Besides, a total of 48 experimental runs have been accomplished using full-factorial design. Thus, the present manuscript can be reorganized as (i) investigation of surface roughness and productivity aspects (ii) platform where the applicability of an advanced multi-objective optimization technique has been investigated, considering HSM technology.

## 2. Experimentation and Optimization Approach

**Figure 1:**
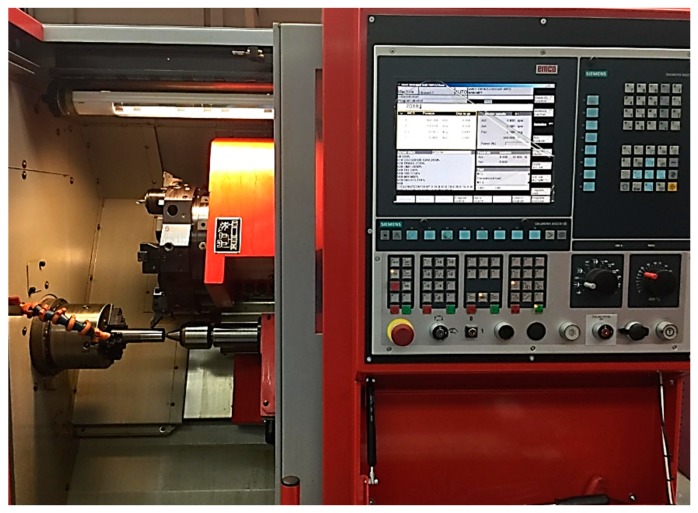
The experimental setup view.

The material used in this paper is “titanium–6aluminum–4vanadium” Grade-5. This alloy is the most popular of the titanium alloys. It is used for a wide range of applications such as in the aerospace, marine, power generation, and offshore industries. Table 1 shows the chemical composition and Table 2 shows the mechanical properties of this alloy. Besides, the CNC turning machine “Emco Concept Turn 45” (Austria), fitted with a Sinumeric 840-D digital NC system (Germany) was used for machining the workpieces. The machining of all test specimens was conducted through a CNC part program. The codes for tool holder and inserts (Sweden) are SVJCL2020K16 and VBMT160404-VBMT331-PM, respectively. The specifications of inserts maintained are clearance angle 7°, cutting edge angle 75°, and nose radius 0.4, and rake angle 6°. All experiments were conducted with cutting fluid conditions using a cooling pump (2.2 KW). The test ring for machining the workpieces is shown in Figure 1. The design variables included in this work are: Cutting speed (i.e., 100 m/min, 200 m/min, and 300 m/min), feed rate (i.e., 0.05 mm/rev and 0.15 mm/rev), depth of cut (i.e., 0.1 mm and 0.3 mm), and cutting length (i.e., 5 mm, 40 mm, 80 mm, and 120 mm). The levels associated with each design variable have been set based on the recommended ranges for the used cutting tool as well as the recommended values in the open literature.

**Figure 2:**
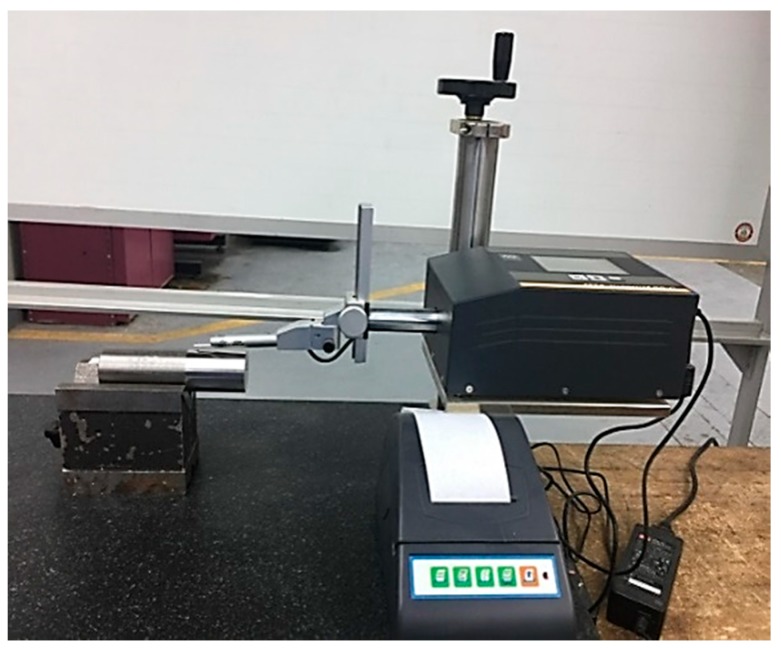
The surface roughness measurements.

A surface roughness apparatus Tesa-Rougosurf-90 G (Switzerland) was used to measure the surface roughness values R_a_, and R_q_, where R_a_ is the arithmetic average of the absolute values of the roughness profile ordinates, and R_q_ is the root mean square average of the roughness profile ordinates. The used settings parameters for surface roughness measurements were: Cut of length of 0.8 mm, cut-off number of 4, measuring speed of 1 mm/s, and using an option of “curved measurement surface”. The test rig for measuring the surface roughness is shown in Figure 2. Regarding the material removal rates, these values are determined using the commonly known formulation based on the speed, feed rate, and depth of cut values. It should be stated that the same insert was used for the same cutting conditions at different cutting lengths.

**Figure 3:**
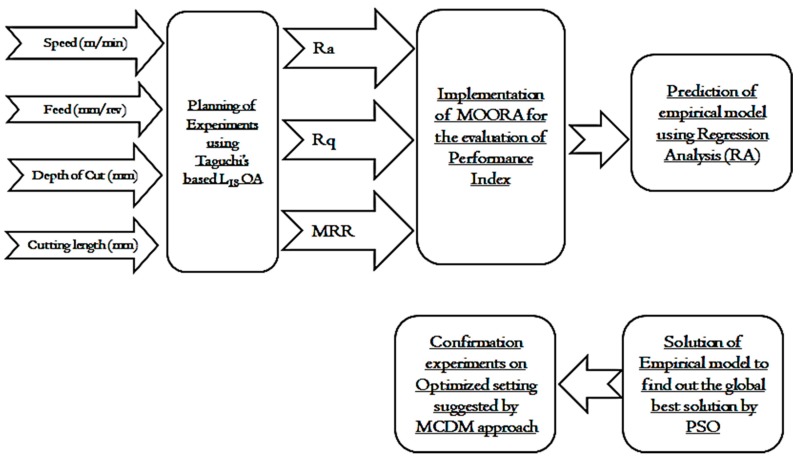
Optimization methodology used in the current research work.

MOORA-regression-PSO-based MCDM approach is shown in Figure 3. The process flow diagram reveals that all the responses converted into performance index (Pi). This Pi is further solved by the empirical model and PSO-based algorithm.

Data normalization using MOORA: The multi-criteria decision-making method by multi-objective optimization with application of the ratio analysis (MOORA) introduced by Brauers [21]. It is the method by which all the response variables were normalized and set in between 0 and 1. After the normalization of response variables all the responses are summed-up and converted into one response known as the performance index (Pi). With the help of the performance index of the MOORA method the complex problems related to multi-objectives can easily be solved. Especially, the problems associated with the responses which are conflicting in nature. By the use of this method, complex decision-making problems in manufacturing can be solved, especially where the response characteristics are of conflicting nature. Following are the steps in order to apply MOORA:

- Step 1: Initially, the input parameters and performance characteristics were defined.
- Step 2: The data should be transformed into a matrix form (decision matrix) as provided by Equation (1):
  (1) $$D=\left[\begin{array}{cccc} D_{11} & D_{12} & . & D_{1n}\\ D_{21} & D_{22} & . & D_{2n}\\ . & . & . & .\\ D_{m1} & D_{m2} & . & D_{\mathrm{mn}} \end{array}\right]$$
  where m and n are the number of alternatives and number of attributes, respectively. In the newly developed ratio system, the comparison is made for the denominator and the performance of the alternative on the attribute. The denominator used in this step is the characteristic of all alternatives for an attribute.
- Step 3: The ratio can be investigated using Equation (2). The square root of the sum of square for each alternative is to be the best choice for the denominator.
  (2) $$D_{\mathrm{ij}}^{*}=\frac{D_{\mathrm{ij}}}{\sqrt{\left[\sum_{i=1}^{m}D_{\mathrm{ij}}^{2}\right]}}$$

It should be stated that the value of D_ij_ varies between 0 to 1 and is a dimensionless quantity. It represents the performance of i_th_ alternative and j_th_ attribute.

MOORA-regression-PSO: The following steps are adopted for the implementation of MOORA-regression-PSO hybrid approach to optimizing the machining parameters for titanium:

- Step 1: In the first step, the data obtained from the experiments were analyzed.
- Step 2: In the second step, the response characteristics were normalized to investigate the performance indices using MOORA.
- Step 3: The statistical analysis of the performance index was made by MiniTab statistical software.
- Step 4: In the next step, the regression coefficients with empirical models were investigated. The empirical model of Pi varies the Pi with the input process variables.
- Step 5: In the fifth step, the empirical model was solved varying the population and generation using PSO. With the implementation of PSO, the global best velocity and positions were determined.
- Step 6: Finally, confirmation experiments were performed at the machining setting suggested by the MCDM approach to analyze the performance characteristics of machining for titanium.

## 3. Results and Discussions: Analysis, Optimization and Validation

Table 3 shows the response characteristics corresponding to different settings of input process parameters. Forty-eight experiments were performed following rules of randomness. It can be observed from Table 3 that both feed rate and depth of cut showed a significant effect on R_a_ and R_q_ values. The performance indices of the reported responses are evaluated, and the results are depicted in Table 4 based on the methodology explained in Section 2 (i.e., constructing the decision matrix and performing the normalization step using ratio analysis). Afterwards, the response characteristics can be combined and converted into a single response termed as the performance index. The MOORA-regression-PSO-based MCDM approach is ready to be employed for multiple performance characteristics optimization. The response characteristics reported in the present work are R_a_ (i.e., lower the better), R_q_ (i.e., lower the better), and MRR (i.e., higher the better) and they are contradictory in nature.

During the implementation of the hybrid optimization approach, the data were converted into the decision matrix using Equation (1). The decision matrix was normalized using Equation (2). After the normalization, the performance indices were evaluated by combining the normalized values of response characteristics corresponding to each experimental run. For example, experiment #1, the Pi: 0.1322 can be calculated by adding the normalized R_a_, normalized R_q_ and normalized MRR (i.e., see in Table 4, column 7 (Pi) = column 4 (normalized R_a_) + column 5 (normalized R_q_) + column 6 (normalized MRR)). Then, the regression analysis was performed to find out the empirical model between the Pi and the input process variables. Statistical analysis of the input process variables along with the investigations of regression coefficients were used in the regression analysis approach. In the present work, a two-factor interaction-like model was obtained after the analysis. Analysis of variance (ANOVA) for the regression analysis is provided in Table 5. It is clear from Table 5 that the statistical analysis reveals a significant regression model (P-value < 0.05). The confidence interval was kept 95% with a P-value less than 0.05. It is evident from Table 5 that the P-value of speed and DoC are greater than 0.05, however, these two parameters were still kept in the model for analysis purposes. The main reason for this consideration is the hierarchical model, in which the terms itself are non-significant, but the interaction of terms plays an important role in the analysis. In the present research, the interaction of speed with DoC, and speed with F shows P-value less than 0.05.

It is evident from Table 5 that the P-value of speed and DoC are greater than 0.05, still these two parameters were kept in the model for analysis purposes. The main reason for this consideration is the hierarchical model, in which the terms themselves are non-significant but the interaction of terms plays an important role in the analysis. In the present research, the interaction of speed and DoC, and speed and F show P-value less than 0.05. Thus, these two parameters in the model provide better analysis. In addition, the contribution percentage of each process parameter on the Pi was investigated. In terms of the contribution percentage results, F (51.4%) has the maximum influence followed by cutting length (4.51%), speed (1.63%), and DoC (0.16%).

The empirical model generated by regression analysis (see Equation (3)) is solved by PSO. The empirical model is affected by certain constraints (i.e., the lower and upper limits of process parameters) as following:

100 ≤ Speed ≤ 300 0.1 ≤ DoC ≤ 0.3 0.05 ≤ Feed≤ 0.15 5 ≤ Cutting Length ≤ 120 Performance Index = 0.12 − 0.0005 × speed − 0.79 × DoC + 3.218 × F + 0.0012 × Cutting Length + 0.0026 × speed × DoC + 0.0046 × speed × F(3)

**Figure 4:**
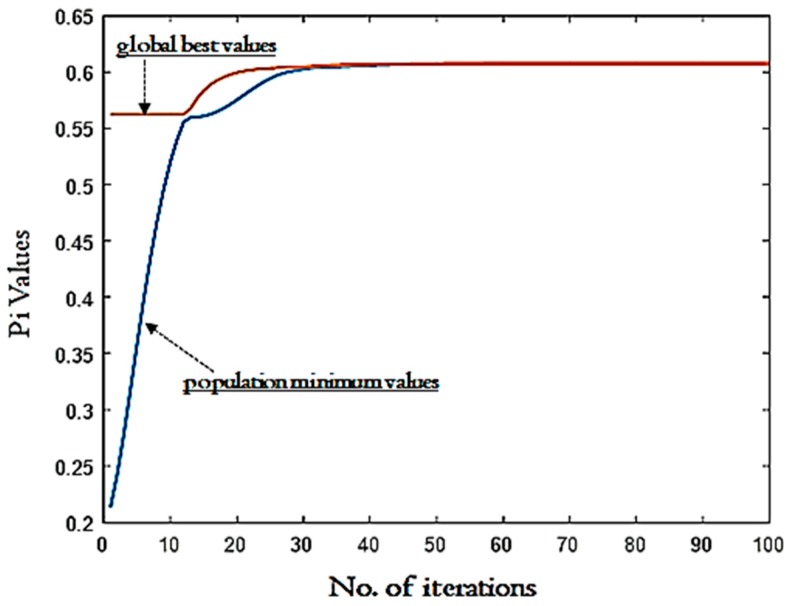
Pi values versus no. of iterations: Convergence analysis.

**Figure 5:**
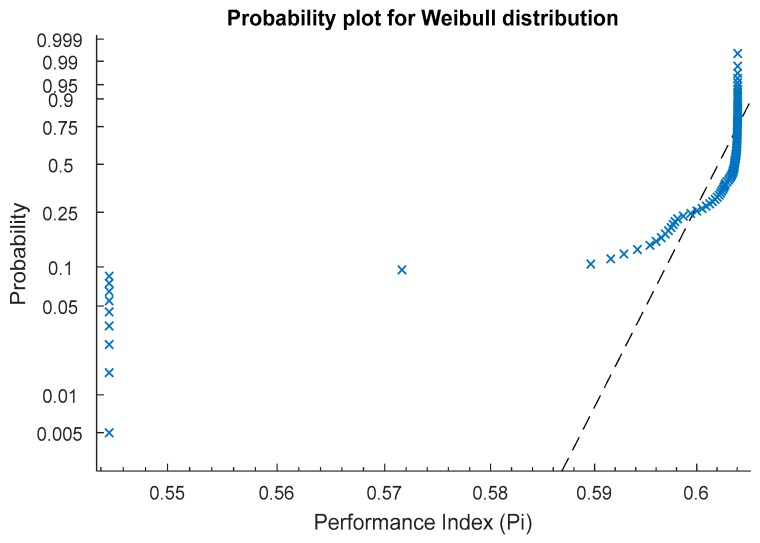
Probability distribution (Weibull) of the optimized Pi values.

In addition, the inertia weight is also selected in the range of 0.4 to 1. Other criteria required for the calculation of optimized setting for Pi values are the acceleration coefficients (i.e., C1 (i.e., cognitive parameter) and C2 (i.e., social parameter) as 1.35 and 2.45, respectively). These coefficients are required to evaluate the response in the search space more efficiently. The basic equations to calculate the values of C1 and C2 are the iteration value dependents. These values vary between 1 and 4 depending on the population, generation, computation complexity. These acceleration coefficients along with the inertia weight help to enhance the efficiency and control the converge rate of PSO. The output predicted using PSO depicts the two best solutions, one refers to the own best and the other refers to the global best. With the increase in the number of iterations, generation and population, the best solution is obtained. In the present work, the optimized value of Pi suggested by PSO is 0.6074. Figure 4 shows the convergence that occurred until reaching the optimized Pi value. It is evident from Figure 4 that after 30 iterations the global best solution was obtained. In Figure 4, the convergence between the population minimum value and global best value takes place. When both lines intersect, the best solution is obtained which is 0.6074 in the current case. Figure 5 of Weibull distribution also verifies the results of the global best solution. The position of global best suggested by PSO is cutting speed 194 m/min, depth of cut of 0.1 mm, feed rate of 0.15 mm/rev, and cutting length of 120 mm. It should be stated that this solution strikes a balance between achieving lower surface roughness in terms of R_a_ and R_q_, with reaching the highest possible material removal rate. Table 6 shows a comparison between the optimal solutions in three cases:

- Minimizing R_a_ and R_q_
- Maximizing MRR
- Multi-objective optimization between R_a_, R_q_ and MRR

It can be found that employing cutting speed (S) 100 m/min, depth of cut (DoC) of 0.1 mm, feed (F) of 0.05 mm/rev, and cutting length (CL) of 5 mm provides the optimal performance in terms of R_a_ and R_q_ (i.e., (S)_100_(DoC)_0.1_ (F)_0.05_(CL)_5_). While achieving the highest MRR was found at run #30 which was performed at cutting speed 300 m/min, depth of cut of 0.3 mm, feed rate of 0.15 mm/rev, and cutting length of 40 mm. In addition, a confirmation test was performed at the suggested optimized settings by the employed hybrid optimization approach. The results were in good agreement with the predicted results as shown in Table 6. It should be stated that the optimized index obtained from the hybrid approach (i.e., 0.607) was not that high, and that is mainly because the approach tries to strike a balance between the two main cutting aspects (i.e., roughness and productivity).

**Figure 6:**
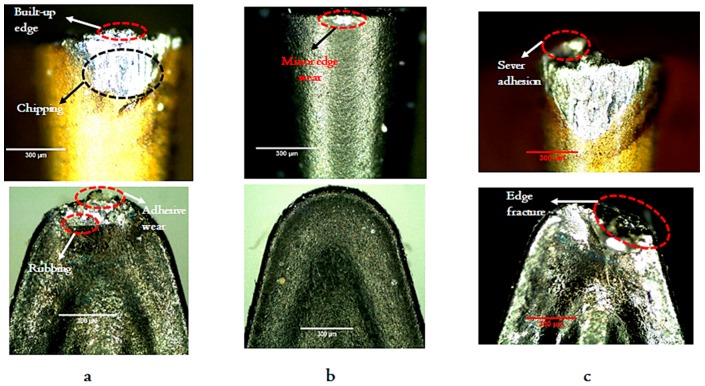
Analysis of tool wear mechanisms: (**a**) Optimized based scenario, (**b**) surface roughness-based scenario, (**c**) MRR based scenario.

The most common tool wear patterns observed under high-speed machining are adhesive, abrasive, micro-chipping, plastic deformation, and built-up edge formations. Mostly, wear mechanism highly depends upon cutting conditions, tool type, and type of workpiece material. In this experimentation, the three studied cases (i.e., surface-quality-based (a), productivity-based (b), and optimized scenario) were explored based on tool wear. In terms of the case (a), Figure 6b shows that the surface-quality-based offered minor edge wear and that is mainly due to employing lower levels of cutting speed, feed rate, depth of cut, as well as the cutting length. However, in the productivity-based scenario, sever tool-chip adhesion and edge fracture were observed (see Figure 6c), and that is mainly due to the high cutting speed and depth of cut values which led to high pressure in the tool-contact area. In terms of the optimized approach (case c), however, it offers a balance between achieving acceptable surface roughness and material removal rate, it shows some disadvantages in terms of tool wear. As can be seen in Figure 6a, chipping, adhesive wear, and built-up edge were still observed. It is mainly due to the high cutting speed compared to the surface roughness-based case. The application of high speed can again lead to high-pressure contact and high generated heat in the cutting zone. However, the tool wear results were not as severe as the productivity-based case. Thus, it is recommended in the future work to establish a detailed multi-objective optimization model which combines surface roughness, MRR, tool wear, machining cost and sustainability aspects as previously discussed in the open literature [22,23] in order to accurately judge the overall machining performance.

## 4. Conclusions and Future Work

Optimization of high-speed machining responses of Ti–6Al–4V has been investigated in the present study using a hybrid approach of multi-objective optimization based on ratio analysis (MOORA) integrated with regression and particle swarm approach (PSO). This optimization approach was employed to offer a balance between achieving better surface quality with maintaining an acceptable material removal rate level. The position of global best suggested by the hybrid optimization approach was: Cutting speed 194 m/min, depth of cut of 0.1 mm, feed rate of 0.15 mm/rev, and cutting length of 120 mm. It should be stated that this solution strikes a balance between achieving lower surface roughness in terms of R_a_ and R_q_, with reaching the highest possible material removal rate. It can be found that employing cutting speed 100 m/min, depth of cut of 0.1 mm, feed rate of 0.05 mm/rev, and cutting length of 5 mm provides the optimal performance in terms of Ra and Rq. While achieving the highest MRR was found at run #30 which has been performed at cutting speed 300 m/min, depth of cut of 0.3 mm, feed rate of 0.15 mm/rev, and cutting length of 40 mm. In addition, a confirmation test was performed at the suggested optimized settings by the employed hybrid optimization approach. The results were in good agreement with the predicted results, as shown in Table 6. It should be stated that the optimized index obtained from the hybrid approach (i.e., 0.607) is not that high, and that is mainly because the approach tries to strike a balance between two main objectives. Finally, an investigation of the tool wear mechanisms for three studied cases (i.e., surface roughness based, productivity based, optimized case) is presented to discuss the effectiveness of each scenario from the tool wear perspective. In terms of the optimized approach (case c), however, it offers a balance between achieving acceptable surface roughness and material removal rate, it shows some disadvantages in terms of tool wear. As can be seen in Figure 6a, chipping, adhesive wear, and built-up edge were still observed. It is mainly due to the high cutting speed compared to the surface roughness-based case. The application of high speed can again lead to high-pressure contact and high generated heat in the cutting zone. However, the tool wear results were not as severe as the productivity-based case. Thus, it is recommended in the future work to establish a detailed multi-objective optimization model which combines surface roughness, MRR, tool wear, as well as machining cost in order to accurately judge the overall machining performance.

## Figures and Tables

**Table 1:** The material composition in percentage form for Ti–6Al–4V.

N	C	H	Fe	O	Al	V	Ti
0.05%	0.1%	0.012%	0.4%	0.2%	6%	4%	Balance

**Table 2:** Mechanical properties of Ti–6Al–4V.

Tensile Strength (MPa)	Yield Strength, 0.2% Offset (MPa)	Elongation (%)	Reduction of Area (%)	Hardness
895	825	10	25	HRC 36

**Table 3:** Experimental results for Ra, Rq, and MRR.

Test #	Speed (m/min)	Depth of Cut (mm)	Feed (mm/rev)	Cutting Length (mm)	R_a_ (µm)	R_q_ (μm)	MRR (mm^3^/min)
1	300	0.1	0.05	5	0.590	0.707	1500
2	300	0.1	0.05	40	0.767	0.866	1500
3	300	0.1	0.05	80	1.048	1.210	1500
4	300	0.1	0.05	120	1.183	1.335	1500
5	300	0.1	0.15	5	2.001	2.459	4500
6	300	0.1	0.15	40	2.539	3.062	4500
7	300	0.1	0.15	80	2.917	3.553	4500
8	300	0.1	0.15	120	4.326	5.213	4500
9	200	0.1	0.05	5	0.470	0.598	1000
10	200	0.1	0.05	40	0.486	0.603	1000
11	200	0.1	0.05	80	0.601	0.758	1000
12	200	0.1	0.05	120	0.689	0.916	1000
13	200	0.1	0.15	5	1.497	1.762	3000
14	200	0.1	0.15	40	1.539	1.883	3000
15	200	0.1	0.15	80	1.808	2.064	3000
16	200	0.1	0.15	120	2.262	2.520	3000
17	100	0.1	0.05	5	0.244	0.310	500
18	100	0.1	0.05	40	0.295	0.392	500
19	100	0.1	0.05	80	0.302	0.384	500
20	100	0.1	0.05	120	0.370	0.455	500
21	100	0.1	0.15	5	1.747	2.264	1500
22	100	0.1	0.15	40	1.869	2.254	1500
23	100	0.1	0.15	80	1.910	2.447	1500
24	100	0.1	0.15	120	2.146	2.502	1500
25	300	0.3	0.05	5	0.687	0.813	4500
26	300	0.3	0.05	40	0.722	0.818	4500
27	300	0.3	0.05	80	0.810	0.935	4500
28	300	0.3	0.05	120	1.031	1.212	4500
29	300	0.3	0.15	5	1.701	1.997	13,500
30	300	0.3	0.15	40	1.877	2.213	13,500
31	300	0.3	0.15	80	4.700	5.352	13,500
32	300	0.3	0.15	120	4.956	6.240	13,500
33	200	0.3	0.05	5	1.008	1.482	3000
34	200	0.3	0.05	40	1.104	1.529	3000
35	200	0.3	0.05	80	1.430	1.728	3000
36	200	0.3	0.05	120	1.533	1.901	3000
37	200	0.3	0.15	5	1.821	2.300	9000
38	200	0.3	0.15	40	1.891	2.355	9000
39	200	0.3	0.15	80	2.002	2.235	9000
40	200	0.3	0.15	120	2.576	2.808	9000
41	100	0.3	0.05	5	0.440	0.533	1500
42	100	0.3	0.05	40	0.633	0.761	1500
43	100	0.3	0.05	80	0.923	1.183	1500
44	100	0.3	0.05	120	0.993	1.218	1500
45	100	0.3	0.15	5	1.452	1.802	4500
46	100	0.3	0.15	40	1.633	1.911	4500
47	100	0.3	0.15	80	1.727	1.999	4500
48	100	0.3	0.15	120	1.788	2.110	4500

**Table 4:** Evaluation of performance index (Pi) using multi-objective optimization based on ratio analysis (MOORA).

Test #	Ra^2^	Rq^2^	MRR^2^	Normalized R_a_	Normalized R_q_	Normalized MRR	Performance Index (Pi)
1	0.3481	0.4998	2,250,000	0.0461	0.0460	0.0401	0.1322
2	0.5883	0.7500	2,250,000	0.0599	0.0563	0.0401	0.1563
3	1.0983	1.4641	2,250,000	0.0819	0.0787	0.0401	0.2007
4	1.3995	1.7822	2,250,000	0.0924	0.0869	0.0401	0.2193
5	4.0040	6.0467	20,250,000	0.1563	0.1600	0.1203	0.4365
6	6.4465	9.3758	20,250,000	0.1983	0.1992	0.1203	0.5178
7	8.5089	12.6238	20,250,000	0.2278	0.2312	0.1203	0.5793
8	18.7143	27.1754	20,250,000	0.3379	0.3392	0.1203	0.7973
9	0.2209	0.3576	1,000,000	0.0367	0.0389	0.0267	0.1023
10	0.2362	0.3636	1,000,000	0.0380	0.0392	0.0267	0.1039
11	0.3612	0.5746	1,000,000	0.0469	0.0493	0.0267	0.1230
12	0.4747	0.8391	1,000,000	0.0538	0.0596	0.0267	0.1401
13	2.2410	3.1046	9,000,000	0.1169	0.1146	0.0802	0.3117
14	2.3685	3.5457	9,000,000	0.1202	0.1225	0.0802	0.3229
15	3.2689	4.2601	9,000,000	0.1412	0.1343	0.0802	0.3557
16	5.1166	6.3504	9,000,000	0.1767	0.1639	0.0802	0.4208
17	0.0595	0.0961	250,000	0.0191	0.0202	0.0134	0.0526
18	0.0870	0.1537	250,000	0.0230	0.0255	0.0134	0.0619
19	0.0912	0.1475	250,000	0.0236	0.0250	0.0134	0.0619
20	0.1369	0.2070	250,000	0.0289	0.0296	0.0134	0.0719
21	3.0520	5.1257	2,250,000	0.1365	0.1473	0.0401	0.3238
22	3.4932	5.0805	2,250,000	0.1460	0.1466	0.0401	0.3327
23	3.6481	5.9878	2,250,000	0.1492	0.1592	0.0401	0.3485
24	4.6053	6.2600	2,250,000	0.1676	0.1628	0.0401	0.3705
25	0.4720	0.6610	20,250,000	0.0537	0.0529	0.1203	0.2268
26	0.5213	0.6691	20,250,000	0.0564	0.0532	0.1203	0.2299
27	0.6561	0.8742	20,250,000	0.0633	0.0608	0.1203	0.2444
28	1.0630	1.4689	20,250,000	0.0805	0.0789	0.1203	0.2797
29	2.8934	3.9880	182,250,000	0.1329	0.1299	0.3608	0.6236
30	3.5231	4.8974	182,250,000	0.1466	0.1440	0.3608	0.6514
31	22.0900	28.6439	182,250,000	0.3671	0.3482	0.3608	1.0761
32	24.5619	38.9376	182,250,000	0.3871	0.4060	0.3608	1.1539
33	1.0161	2.1963	9,000,000	0.0787	0.0964	0.0802	0.2553
34	1.2188	2.3378	9,000,000	0.0862	0.0995	0.0802	0.2659
35	2.0449	2.9860	9,000,000	0.1117	0.1124	0.0802	0.3043
36	2.3501	3.6138	9,000,000	0.1197	0.1237	0.0802	0.3236
37	3.3160	5.2900	81,000,000	0.1422	0.1496	0.2405	0.5324
38	3.5759	5.5460	81,000,000	0.1477	0.1532	0.2405	0.5415
39	4.0080	4.9952	81,000,000	0.1564	0.1454	0.2405	0.5423
40	6.6358	7.8849	81,000,000	0.2012	0.1827	0.2405	0.6244
41	0.1936	0.2841	2,250,000	0.0344	0.0347	0.0401	0.1091
42	0.4007	0.5791	2,250,000	0.0494	0.0495	0.0401	0.1390
43	0.8519	1.3995	2,250,000	0.0721	0.0770	0.0401	0.1891
44	0.9860	1.4835	2,250,000	0.0776	0.0792	0.0401	0.1969
45	2.1083	3.2472	20,250,000	0.1134	0.1172	0.1203	0.3509
46	2.6667	3.6519	20,250,000	0.1276	0.1243	0.1203	0.3721
47	2.9825	3.9960	20,250,000	0.1349	0.1301	0.1203	0.3852
48	3.1969	4.4521	20,250,000	0.1397	0.1373	0.1203	0.3972

**Table 5:** Analysis of variance (ANOVA) for the regression model: Pi analysis.

Source	Degree of Freedom (DF)	Statistical Summation (SS)	Contribution Percentage (%)	Mean Square (MS)	F-Value	P-Value
Model	11	2.34519		0.21320	20.98	0.000
Speed	2	0.04423	1.63	0.02211	2.18	0.128
DoC*	1	0.00427	0.16	0.00427	0.42	0.521
F*	1	1.39348	51.4	1.39348	137.11	0.000
Cutting Length	3	0.12240	4.51	0.04080	4.01	0.015
Speed*DoC	2	0.68923	25.42	0.34462	33.91	0.000
Speed*F	2	0.09158	3.38	0.04579	4.51	0.018
Error	36	0.36586	13.5	0.01016		
Total	47	2.71106				

* DoC: depth of cut; *F: feed

**Table 6:** A summary of optimizations results and validation.

Cutting Conditions	Initial Machining Parameter		Predicted	Experimental
	R_a_ and R_q_	MRR		
	(S)_100_(DoC)_0.1_(F)_0.05_(CL)_5_	(S)_300_(DoC)_0.3_(F)_0.15_(CL)_120_	(S)_190_(DoC)_0.1_(F)_0.15_(CL)_120_	(S)_190_(DoC)_0.1_(F)_0.15_(CL)_120_
Ra	0.244	4.956	2.262	2.19
R_q_	0.31	6.240	2.52	**2.43**
MRR	500	13,500	3000	2850
Pi	-	-	0.6074	-
